# Efficacy of conservative treatment regimes for hip osteoarthritis - Evaluation of the therapeutic exercise regime "Hip School": A protocol for a randomised, controlled trial

**DOI:** 10.1186/1471-2474-12-270

**Published:** 2011-11-24

**Authors:** Inga Krauss, Benjamin Steinhilber, Georg Haupt, Regina Miller, Stefan Grau, Pia Janssen

**Affiliations:** 1 Medical Clinic, Department of Sports Medicine, University of Tuebingen, Germany

## Abstract

**Background:**

Hip osteoarthritis (hip OA) is a disease with a major impact on both national economy and the patients themselves. Patients suffer from pain and functional impairment in activities of daily life which are associated with a decrease in quality of life. Conservative therapeutic interventions such as physical exercises aim at reducing pain and increasing function and health-related quality of life. However, there is only silver level evidence for efficacy of land-based physical exercise in the treatment of hip OA. The purpose of this randomized controlled trial is to determine whether the specific 12-week exercise regime "Hip School" can decrease bodily pain and improve physical function and life quality in subjects with hip osteoarthritis.

**Methods/Design:**

217 participants with hip OA, confirmed using the clinical score of the American College of Rheumatology, are recruited from the community and randomly allocated to one of the following groups: (1) exercise regime "Hip School", n = 70; (2) Non-intervention control group, n = 70; (3) "Sham" ultrasound group, n = 70; (4) Ultrasound group, n = 7. The exercise regime combines group exercises (1/week, 60-90') and home-based exercises (2/week, 30-40'). Sham ultrasound and ultrasound are given once a week, 15'. Measures are taken directly prior to (M1) and after (M2) the 12-week intervention period. Two follow-ups are conducted by phone 16 and 40 weeks after the intervention period. The primary outcome measure is the change in the subscale *bodily pain *of the SF36 from M1 to M2. Secondary outcomes comprise the WOMAC score, SF36, isometric strength of hip muscles, spatial-temporal and discrete measures derived from clinical gait analysis, and the length of the centre of force path in different standing tasks. An intension-to-treat analysis will be performed using multivariate statistics (group × time).

**Discussion:**

Results from this trial will contribute to the evidence regarding the effect of a hip-specific exercise regime on physical function, pain, and health-related quality of life in patients with hip osteoarthritis.

**Trial registration:**

German Clinical Trial Register DRKS00000651.

## Background

### Prevalence and treatment modalities of osteoarthritis

Osteoarthritis (OA) is a disease with a major impact on both the national economy and the patients themselves. In Germany, approximately five million people suffer from osteoarthritis [1]. It is the most prevalent musculoskeletal disease and its impact on morbidity and mortality in industrialized countries is almost as large as the impact of respiratory or digestive disorders [2]. The knee and hip joints are the most commonly affected joints of this disease [1,3]. Data from a large cohort-study reveal hip symptoms in 36% of an American population aged 45 and older. 28% of this population had radiographic, and 10% had symptomatic hip OA [4]. Prevalence for symptomatic hip OA increases in older individuals and women [4]. According to demographic estimates, more than 20% of the population will be older than 65 years in 2040 [5], and the impact of OA will further increase. Patients suffer from pain and functional impairment in activities of daily life such as walking or climbing stairs [6,7]. Strength deficits and gait disturbances like gait asymmetries and reduced walking speed are frequent findings [8-10]. Bodily pain and deficits in physical functioning are associated with a decrease of life quality [7,11].

Joint replacement is the treatment strategy in the final stages of hip or knee OA. In earlier stages, conservative therapeutic interventions are important to reduce pain and increase function and health-related quality of life [12]. In this respect, physical therapy and physical exercise programs are relevant and important therapeutic options. Here, treatments during which the patient is active are preferable to passive therapies [13,14]. Physical exercises in particular are non-hazardous in terms of side-effects and can have positive effects on life quality, management of everyday life and physical functioning [15-19]. Exercises can further decrease pain and their efficacy was shown to be comparable to the efficacy of non-steroidal anti-inflammatory drugs (NSAIDs). In addition, they do not have expected adverse reactions in comparison to the long term use of NSAIDs [14,20].

### Evidence for effectiveness of exercises in the treatment of lower limb osteoarthritis

Platinum/1a level of evidence is given for the efficacy of joint related exercise programs with respect to pain reduction and increase of function in subjects with knee OA. It applies to analyses including data of aquatic and land-based exercises, as well as for land-based exercises only [21,22]. According to the EULAR evidence based recommendations for the management of hip osteoarthritis published in 2004, data on exercise interventions for hip OA are required to determine the benefit of treatments at each key site of OA, as there may be true treatment differences for OA according to the site affected [23]. More recently, meta-analyses have been published focusing explicitly on the effects of exercise on hip osteoarthritis. Combined data of aquatic and land-based exercises for hip OA demonstrate evidence (1a) for the reduction in pain [24,25]. However, there is only silver-level evidence for the efficacy of land-based physical exercise to reduce pain and to improve function in the treatment of hip OA [26]. The aforementioned review summarizes the effects of only five randomized controlled trials (RCTs) which were included into the meta-analysis. Only one of these studies explicitly focused on patients with hip osteoarthritis. The authors conclude that the limited number and small sample size of the included RCTs restricts the confidence that can be attributed to the presented results. Therefore, adequately powered RCTs evaluating exercise programs specifically designed for people with symptomatic hip OA need to be conducted [26].

Aside from a lack of large RCTs studies on subjects with hip OA, there is not much known about how an optimised training program should be designed and what kind of training would be the most efficient for treating hip and knee OA [19,27,28].

### Hip School

The group exercise program "Hip School" was established in 1996 [29,30]. Since then, more than 20 therapy groups have been organized in the region allowing many patients with hip osteoarthritis access to a supervised training program. Several studies were conducted previously to evaluate the efficacy of the Hip School [31-34]. However, its efficacy has not yet been demonstrated in a randomised controlled trial using generally accepted measures. This lack of evidence is being addressed by the clinical trial described in this study protocol. The protocol is based on (a) previous pilot studies investigating the reliability, validity, and feasibility of the methods that will now be used to quantify efficacy outcomes in subjects with hip osteoarthritis [35-37], and (b) an interventional study used to quantify efficacy and feasibility of an additional home-based exercise program in combination with the aforementioned therapy regime [38-40].

### Study purpose

The primary aim of this study is to determine whether a 12-week intervention program comprising group and home-based exercises decreases bodily pain in subjects with hip osteoarthritis in comparison to a non-treated control group. Efficacy is quantified by the subscale *bodily pain *of the SF36. The secondary aim is to determine whether this exercise regime can improve function, health-related quality of life, strength of hip surrounding muscles, postural control in bi-pedaled, tandem-, and single leg stance, joint function, and gait parameters in comparison to a non-treated control group, an attention control group (sham ultrasound), and an ultrasound group. Ultrasound therapy is only investigated in an explorative manner. Follow-up data are recorded to evaluate the sustainability of the interventional programs over another 40 weeks.

It is hypothesized that a 12-week intervention program will (1) decrease pain, and (2) improve physical function in comparison to a non-treated control. It is further hypothesized that (3) the efficacy of the exercise regime is superior to the efficacy of an intervention solely aiming for attention control, and (4) that effects are long-acting.

## Methods/Design

### Design

**Figure 1:**
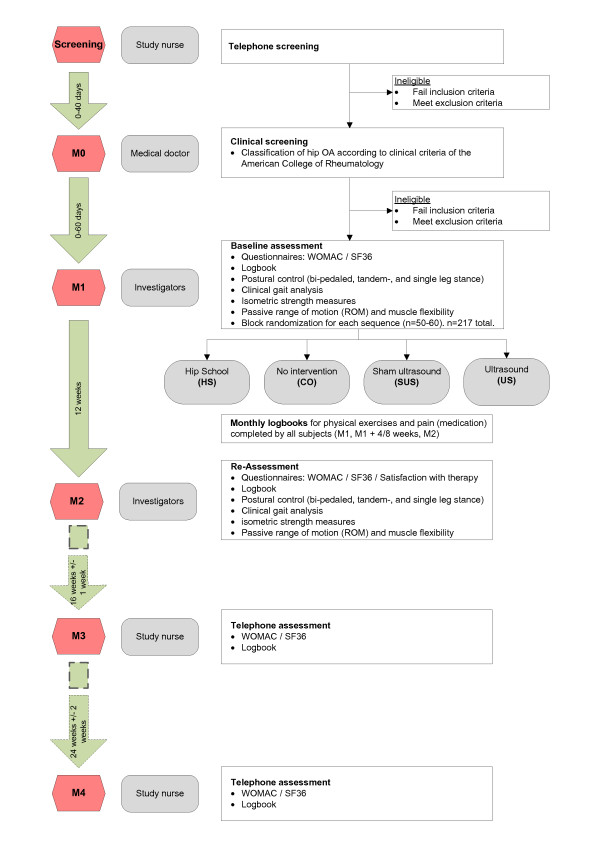
**Trial protocol**.

This is a randomized, controlled trial, single-blinded for participants for two of the four treatment options (sham ultrasound and ultrasound) (Figure 1). Recruited patients undergo telephone screening followed by a clinical examination by a medical doctor to ensure eligibility. Eligible participants then undergo outcome assessment using the complete test battery prior to intervention, and will subsequently be randomized to one of the four groups. A ratio of 10:10:10:1 will be used for randomization into Hip School (HS), no intervention group (CO), sham ultrasound group (SUS), and ultrasound group (US). During the 12 week intervention period, monthly logbooks for physical exercises and pain have to be completed by all subjects. The complete test battery is repeated after the 12 week interventional period. Questionnaires (WOMAC, SF36) and log books are accessed via telephone assessment 16 and 40 weeks after the retest.

### Randomization process and allocation concealment

Randomization is stratified by gender due to its possible influence on efficacy of strengthening exercises [41,42]. Since not all subjects can be measured and treated simultaneously, the study is divided into four identical interventional periods (sequence 1-4), and the mentioned allocation procedure is performed in permuted blocks of 50-60 subjects each. The randomization sequence is generated electronically prior to each interventional period. Participants draw a lot with a randomization number at the beginning of baseline assessment. This process is double-blinded (participant and investigator). At the end of the baseline assessment, a study nurse compares the randomization number of the lot with the randomization list displaying information on treatment allocation. Group allocation is then documented on an extra sheet of the case report form. The case report form is filed after baseline assessment. The assignment process as described prevents foreknowledge of treatment assignment.

### Blinding

Treatment allocation to ultrasound and sham-ultrasound is blinded to the subject. Blinding of the investigator is not possible as sham-ultrasound is applied according to the normal routine without turning on the power.

Day and Altman suggest blinding assessment by using non-involved assessors to record outcome variables [43]. However this is not feasible in the context of the given study. To allow readers to judge possible information bias, assessment routine and blinding are stated explicitly [44]. Blinding is impossible as treatment exposure can be discovered by interviewing the study participants (except the differentiation between US and SUS, which is kept single-blinded until the end of the study). Furthermore, independent assessors are not available, as quantification of outcomes needs specific know-how and man-power of our institution is limited. Moreover, instrumented gait analysis and assessment of postural control using pressure mats are relatively objective measures with limited possibility of exerting external influence. Strength measures may be more susceptible to external influences, as motivation is an important factor for maximum strength development. To minimize potential influence of the assessors, instructions are standardized and always given by the same investigator. SF36 and WOMAC are self-administered psychometric instruments. Subjects are introduced to the questionnaires at the beginning of the baseline assessment before treatment allocation is done. Subjects may contact the study nurse in case of questions. However, there is no assessor recording the responses of the subjects to the questionnaires and therefore no need for blinding.

### Participants

217 men and women aged between 18 and 85 years are recruited via regional press, flyers in medical and physiotherapeutic practices, the outpatient clinic of the Department of Sports Medicine, and personal communication. Eligibility is confirmed by verbal and clinical examination carried out by the principal investigator of the study (medical doctor). Criteria for hip osteoarthritis are defined according to the clinical criteria of the American College of Rheumatology [45]. Other inclusion criteria are: (i) health-related eligibility in terms of physical and mental ability to participate in the interventional program; (ii) sufficient time to keep therapeutic appointments; (iii) capacity to consent. Annotation: Subjects can also be included in the case of a contra-lateral hip replacement, as long as one side has a hip osteoarthritis according to the above mentioned criteria.

Exclusion criteria are: (i) instable anchoring of an artificial hip replacement; (ii) luxation as an adverse event of artificial hip replacement; (iii) predominant knee OA; (iv) pathologies in the region of the lower extremities or lower back that are not related to OA and need medical treatment; (v) inability to ambulate without walking aids; (vi) hip joint injections within the last 3 months; (vii) surgery at the lower extremity within the last 3 months; (viii) previous trauma at the hip or pelvis with subsequent development of a secondary arthrosis; (vix) known endocrinological cause of hip OA (hyperuricaemia, hyperparathyroidism, hyperuricaemia); (x) verified metabolic cause of hip OA (haemochromatosis, rachitis, chondrocalcinosis, ochronosis); (xi) state after aseptic osteonecrosis (Perthes' disease); (xii) neurological disease leading to sensomotoric deficits; (xiii) cardio-vascular disease or other co-morbidities resulting in a profoundly decreased physical capacity in everyday life and that are known as contraindications for physical activities (i.e. cardiac insufficiency NYHA III-IV, terminal renal insufficiency); (xiv) medical exercise therapy or physiotherapy using weight machines and comparable resources during the last 3 months and carried out at least 6 times; (xv) specific group or individual intervention to address hip OA in the last 3 months (minimum 1x/week, 30 minutes or more); (xvi) physical therapy to address hip OA (minimum 1x/week); (xvii) novel initiated physical exercise within the last 3 months (minimum 1x/week to be short of breath for at least 30 minutes); (xviii) abuse of drugs or alcohol; (xix) participation in another clinical trial in the last four weeks; (xx) no compliance; (xxi) acute illness.

Ethical approval has been obtained from the Ethics Committee of the University of Tuebingen (358/2010BO2). All participants will provide written informed consent.

### Interventions

All participants are requested to refrain from seeking other forms of treatment during the 12 week-intervention period from M1 to M2. Subsequently, subjects are offered to participate in the intervention group they were not allocated to before for another 12 weeks.

Many studies compare exercise interventions with an untreated control group (parallel group design or waiting list design) [26,46]. However it is well known that a placebo is significantly superior to the effect of untreated controls [47]. The used study design will help to distinguish between treatment effects of physical exercise, attention control (sham ultrasound), and unaccompanied study participation (no intervention group).

#### Hip School(HS)

The Hip School program is a comprehensive, pre-defined 12-week training program. Not all exercises can be displayed in detail as this would be beyond the scope of this study protocol. However, some details are listed below to allow an opinion to be made on relevant issues of the training program.

The Hip School comprises group (1x/week, 60-90') and home based exercises (2x/week, 30-40'). Group size is restricted to a maximum of 15 participants. Elastic rubber bands, stability trainers (pads), exercise balls and exercise mats are used as training devices. Group and home training sessions include hip specific elements of motor learning and mobilization, strength training and exercises to improve postural control, as specified below. Group sessions further include education related to exercises, such as information on anatomical basics and training modalities. The sessions enhance social contacts by having group-based introductions and feedbacks before and after the exercises, and by enforcing partner and group exercises. In the group lessons, subjects are introduced to the exercises they have to do at home. In addition, subjects receive exercise leaflets with pictures of the exercises and written instructions every week.

**Figure 2:**
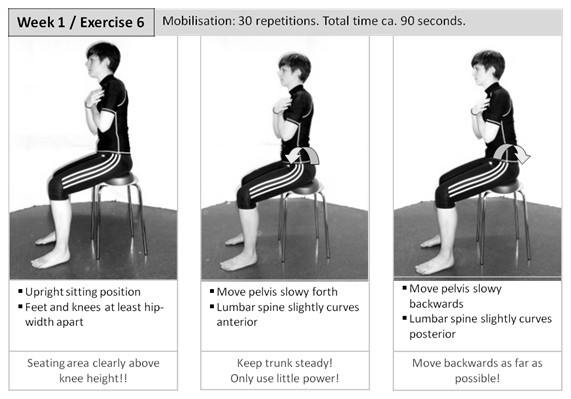
**Mobilisation exercise**. Example of an exercise for lumbar spine, pelvis and hip joint mobilisation.

Detailed description of group and home-based training modalities:

Mobility exercises include elements to increase flexibility of the lumbar spine, pelvis and hip joints. An example for sagittal plane motion is given in Figure 2. Muscle stretching mainly addresses hip and knee flexors and extensors and hip adductors. Perception skills are trained to allow proper exercise execution, and to enhance motor control.

**Figure 3:**
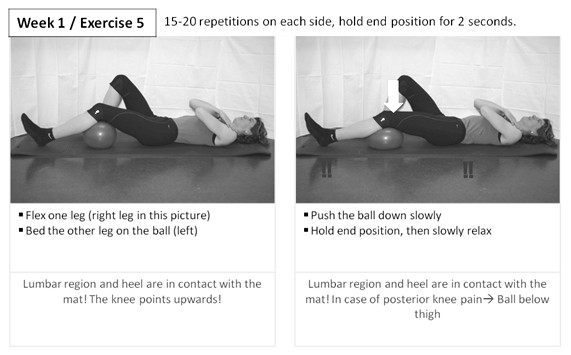
**Strength training for hip and knee extensors**. Example of an exercise for closed kinematic chain training.

**Figure 4:**
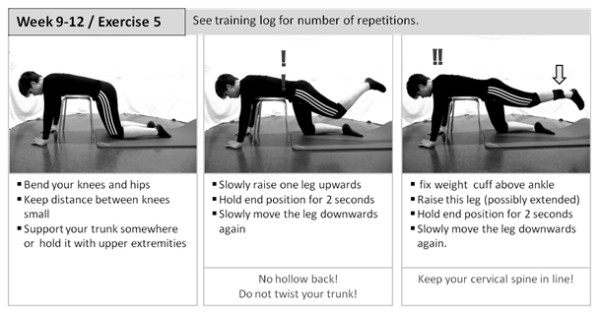
**Strength training for hip extensors**. Example of an exercise for open kinematic chain training.

Strength training comprises open and closed kinematic chain exercises for extensors, flexors, abductors and adductors of the hip, as well as flexors and extensors of the knee. Examples for open and closed kinematic chain exercises for hip extensors are given in Figures 3 and 4. Intensity and structure of the exercises follow a progressive concept (Table 1) and are monitored using a Borg Scale.

**Table 1:** Training progression for strength exercises.

Week	Objective	Intensity (% max strength)	Repetitions/Sets
**1-3**	Motor learning	< 30%	≥ 30/1

**4-8**	Strength endurance	30-40%	20-25/2-3

**9-12**	Endurance & maximum strength	40-70%	10-15/3-4

**Figure 5:**
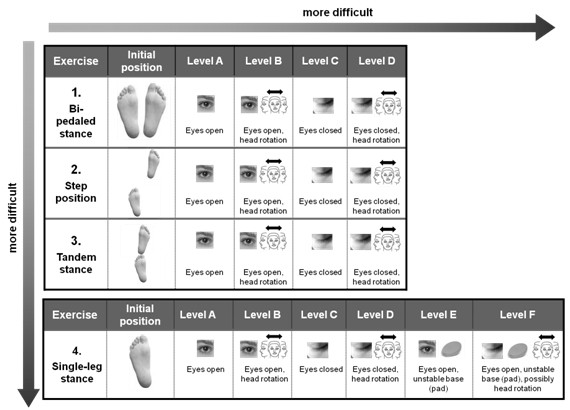
**Progressive training concept**. Procedural method for static exercises of postural control.

Leg-alignment training is included in all exercises for postural control. These exercises also follow a progressive training concept, as displayed in Figure 5 for static conditions. Week 9-12 includes further dynamic exercises.

#### No intervention group (CO)

Subjects allocated to this group do not receive any therapeutic intervention between M1 and M2. They are requested to continue their usual routine.

#### Sham ultrasound (SUS)

Frequency, duration, and total number of treatments for sham ultrasound are equivalent to the procedure described for ultrasound. However, intensity is turned off.

#### Ultrasound (US)

Despite low quality of given evidence, therapeutic ultrasound may be beneficial for patients with osteoarthritis of the knee [48]. In subjects with hip OA, ultrasound in addition to standard physiotherapy has been shown to be superior in terms of a longitudinal positive effect on pain, functional status, and physical quality of life in comparison to a standard physiotherapy with and without sham ultrasound [49]. Safety of this intervention has yet not been disproved, and to date no serious adverse events have been reported [48]. In the context of this study, ultrasound is used as a comparable verum intervention to sham ultrasound (SUS). It would be unethical to include SUS only, as subjects are told that they may receive real ultrasound or sham ultrasound. US will be applied in a minor number of subjects, serving to explore its potential benefits in the treatment of hip OA in a different dosage as investigated before [49]. Ultrasound is applied with a commercially available ultrasound apparatus (Zimmer MedizinSysteme GmbH, Germany). Instrument adjustments and treatment modalities are presented in Table 2.

**Table 2:** Instrument adjustments and treatment modalities for ultrasound therapy.

Parameter	Setup
**Frequency**	1 MHz
**Mode**	Continuous mode
**Intensity**	1 W/cm^2^
**Head size**	5 cm^2^
**Duration of treatment**	15' (5' each from anterior, lateral, and posterior)
**Treatment area**	70-80 cm^2 ^about the affected hip joint
**Treatment frequency**	1/week
**Total number of treatments**	12

### Outcome measures (Figure 1)

Participants are assessed at baseline (M1), and 12 weeks after the intervention period (M2). Patient characteristics (age, gender, height, weight) are evaluated at the baseline assessment. Questionnaires (WOMAC, SF36), measures of postural control, clinical gait analysis, functional tests (passive range of motion (ROM), muscle flexibility), and isometric strength measures are evaluated at M1 and M2. Previous physiotherapy and/or exercises, medication, and pain (retrospective time frame of four weeks) are evaluated at M1 and noted each subsequent month up to M2 in a logbook. Satisfaction with the therapy regime is assessed with a questionnaire at M2 and M3. 16 weeks past M2 (M3) and 24 weeks past M3 (M4), participants are contacted again via telephone to assess WOMAC, SF36, previous physiotherapy and/or exercises, and medication.

#### Questionnaires

The 36-item Short Form (SF36) is a well-established generic health status measure and allows the effect of an OA intervention to be gauged in comparison with other interventions [50]. It comprises different scales, four of them related to physical health (physical functioning, role-physical, bodily pain, general health), and four of them related to mental health (vitality, social functioning, role-emotional, mental health). The primary outcome measure of this study is the subscale bodily pain of the SF36. The other scales and sum scores will also be analysed as secondary outcomes.

The Western Ontario McMasters Universities Osteoarthritis Index (WOMAC^® ^NRS German for Germany 3.1 Index) is a disease-specific instrument used to evaluate self-reported pain, stiffness and functional impairment. It is a valid, reliable and responsive score, easy to complete, simple to score and available in multiple language forms and scaling formats [51].

A five point Likert Scale is used on M2 and M3 to quantify satisfaction with the therapy regime, and whether participants would recommend this therapy to others.

Level of education of the subjects is recorded to control for a recruiting bias.

#### Monthly logbooks

Participants are asked to fill out a monthly logbook between M1 and M2. They have to specify previous physiotherapy and/or exercise, medication, and pain (retrospective time frame of four weeks). This information is also requested on M3 and M4 in the context of the telephone call.

#### Measures of postural control

Postural control is quantified in bi-pedaled, tandem-, and single leg stance on a measuring system for force distribution (FDM System, zebris Medical GmbH, Isny, Germany). The outcome measure for postural control is the total path of the centre of force over the entire trial. Subjects are allowed to familiarize themselves with each mode prior to recording. Modalities of the different tests for postural control are summarized in Table 3. Trials of each mode are averaged prior to further analysis.

**Table 3:** Test modalities for postural control

Test	Position	Recording time	Number of trials
**Bi-pedaled with eyes open**	Feet in parallel position Weight is equally distributed on the feet Knees slightly bended Arms akimbo	10 s each	3 each
**Bi-pedaled with eyes closed**			

**Tandem stance right foot in front**	Feet are placed on a line, one before the other Toes of the rear foot are in contact with the heel of the leading foot Weight is equally distributed on both feet Knees are slightly bended Arms akimbo	6 s each	3 each
**Tandem stance left foot in front**			

**Single-led stance right**	Single led stance Knee of the supporting leg is slightly bended Non-supporting leg is not allowed to touch supporting leg Arms akimbo	6 s each	5 each
**Single-led stance left**			

#### Clinical gait analysis

Participants undergo three-dimensional gait analysis in barefoot and shod conditions at self-selected normal speed that is monitored by light-barriers. After two static trials, participants continuously walk back and forth on an eleven meter walk-way until at least seven valid trials for each side and condition are captured. For the barefoot condition, the walk-way is covered with an EVA-foam (shore 80) to allow a comfortable and natural walking pattern. Kinematic and spatial-temporal data are collected using a Vicon motion analysis system with six cameras (ViconPeak, MCAM M1, 120 Hz, Oxford, UK). Data are analysed with the lower body model of the conventional gait model Plug-in-Gait (Vicon Polygon, Oxford Metrics Ltd., Oxford, UK). Markers are placed on the superior and posterior iliac spines, lateral thigh, and lateral epicondyle of the knee, lateral shank, lateral malleolus, calcaneus, and second metatarsal head. Additional markers are placed on the toe or toe cap, respectively, to ease the detection of toe off.

Outcome measures include the following spatial-temporal variables: stride length, step length, step width, walking velocity, cadence, single and double support time. Other variables of interest include time histories, maximum and minimum joint angles, and ranges of motion of the sagittal plane for pelvis, hip, knee, and ankle joint, as well as pelvic drop and hip adduction and abduction in the frontal plane. Outcome measures of five trials for each limb and condition (barefoot and shod) are extracted and averaged with a customized Matlab routine (Mathworks Inc., Natick, Massachusetts, USA).

#### Functional tests

Pelvic obliquity, passive ranges of motion of the hip, knee and ankle joint and muscular flexibility of the iliopsoas muscle, rectus femoris muscle and the hamstrings are examined by a physiotherapist.

#### Isometric strength measures

The Isomed 2000 (D&R GmbH, Hernau, Germany) isokinetic dynamometer is used to measure isometric peak torque for hip abduction (HAB), hip adduction (HAD), hip flexion (HF) and hip extension (HE). The feasibility and reliability of the test protocol was evaluated in a previous study. It was shown, that HAD and HE are prone to larger measurement errors. This can be explained by a hindered fixation, as the pelvis is pried upwards when the leg pushes downwards [37]. For this reason, tests are accompanied by two investigators to ensure proper fixation and to decrease the time necessary per measurement and ensuring a high level of standardization with respect to the test procedure. While one investigator operates the device and give standardized instructions to each subject, another investigator manually stabilize the subject's pelvis during all measurements of HAD and HE, where the pelvis tends to become instable. This investigator further monitors the subjects during the measurements for signs of pain or muscle cramps.

Test procedure: Subjects accomplish a five minute warm-up on a bicycle ergometer (50-100 Watt) followed by some stretching exercises. Subsequently, the randomly selected starting leg is allotted and subjects remove their shoes. Prior to the test, a knee orthosis is attached to the tested leg. For HAB and HAD, the orthosis is set at 90° at the knee. For HF and HE, the orthosis is set at 0°. The orthosis is necessary to decrease measurement artifacts related to oscillations and resulting moments of inertia of the shank and foot. All test modes are performed on each leg separately. Test mode and sequence, subject positioning, joint angle position and annotations for fixation are given in Table 4. Maximal axis-joint alignment is insured before subjects are fixed to the surface and dynamometer. Each trial is initialised by a sub-maximum test trial to allow participants to get used to the work movement and measurement procedure. Participants are then asked to contract their muscles to the maximum three times in a given time frame of 40 seconds against the fixed lever arm of the dynamometer. Each test trial is followed by a break of 30 seconds. After all strength tests, a physiotherapist performs some traction and stretching exercises to allow relaxation of joints and muscles of the participants.

**Table 4:** Test modalities for isometric strength measures.

Test mode and sequence	Position and fixation	Joint angle
**1. Hip abduction (HAB)**	• Recovery position (lateral) • Belts strapped around pelvis and contra-lateral leg	• 0°
		
**2. Hip adduction (HAD)**	• lower arm on the pillow, upper arm grasps the edge of the examination table • Tested leg in 90° knee flexion, fixed with orthosis	• 20° abduction
		
**3. Hip flexion (HF)**	• Supine position • Belts strapped around pelvis and contra-lateral leg	• 20° flexion
		
**4. Hip extension (HE)**	• Arms are folded • Tested leg in 0° knee flexion, fixed with orthosis	• 60° flexion

### Proposed sample size/power calculation

The primary endpoint is the change of SF36: Subscale bodily pain from M1 to M2 of HS in comparison to CO. Responsiveness of the subscale is comparable to the pain scale of the disease specific WOMAC in patients with osteoarthritis undergoing a rehabilitation intervention [52]. The SF36 is recommended as a generic instrument for recording relevant domains in osteoarthritis of the lower limb [53]. It is the most widely used generic health instrument [52] and allows comparison of disease states within and between different disorders.

Sample size calculation is based on clinically relevant changes: An effect smaller than the minimum clinical important difference (MCID) may be measureable although patients will be unable to notice it [50]. MCID was derived from literature [50], basic values and statistical spread of the measure for the given population were derived from a pilot study of our own research group on subjects with hip osteoarthritis. According to the mentioned presumptions, a sample size of n = 60 in each group will have 80% power to detect a clinically relevant difference in bodily pain for HS in comparison to CO using a two group t-test with a 0.05 two-side significance level. A failure rate of 15-20% is approximated, thus final sample size is set at n = 70 for each group, except US with n = 7. This is only an explorative intervention. Sample size was calculated using G*Power Version 3.0.10 (Franz Faul, Universitaet Kiel, Germany).

### Statistical analysis

Data will be analyzed on an intention-to-treat principle using all randomized participants. Missing data will be filled in by carrying the last score forward. A per protocol analysis will also be conducted as a subordinate type of analysis. Demographic characteristics, joint and muscle function, and logbooks will be displayed with descriptive statistics. Multivariate statistics will be used (factors: group × time) for outcomes measured on a continuous scale. For strength measures, baseline levels will be included as covariates.

## Competing interests

The authors declare that they have no competing interests. Exercise materials are sponsored, yet sponsorship has no influence on design, process, analysis, results or interpretation of the study data.

## Authors' contributions

IK, BS and GH conceived and designed the trial protocol. SG and IK initiated the research project. IK, BS, GH, PJ and RM were involved in the pilot studies that are taken as basis for this study protocol. PJ is the medical investigator of the study. IK drafted the manuscript. All authors read and revised the manuscript critically for important intellectual content. All authors approved the final manuscript.

## Pre-publication history

The pre-publication history for this paper can be accessed here:

http://www.biomedcentral.com/1471-2474/12/270/prepub
